# 3,4-Dimethyl-1-phenyl­pyrano[2,3-*c*]pyrazol-6(1*H*)-one

**DOI:** 10.1107/S1600536811011317

**Published:** 2011-03-31

**Authors:** Neman Ahmad, M. Nawaz Tahir, Misbahul Ain Khan, Abdul Qayyum Ather, Muhammad Naeem Khan

**Affiliations:** a Department of Chemistry, Islamia University, Bahawalpur, Pakistan; b Department of Physics, University of Sargodha, Sargodha, Pakistan; c Applied Chemistry Research Center, PCSIR Laboratories Complex, Lahore 54600, Pakistan

## Abstract

In the title compound, C_14_H_12_N_2_O_2_, the dihedral angle between the phenyl ring and the 3,4-dimethyl­pyrano[2,3-*c*]pyrazol-6(1*H*)-one system is 7.28 (6)°. An intra­molecular C—H⋯O inter­action generates an *S*(6) ring. In the crystal, the mol­ecules are linked by C—H⋯O hydrogen bonds, forming *C*(8) chains. C–H⋯π and π–π inter­actions [centroid–centroid separation = 3.6374 (12) Å] further consolidate the packing.

## Related literature

For a related structure, see: Ramsay & Steel (1985) ▶. For background to the pyrano[2,3-*c*]pyrazol-6-one ring system, see: Abdallah & Zaki (1999 ▶); Huang *et al.* (1992 ▶); Khan *et al.* (1982 ▶); Kuo *et al.* (1984 ▶); Ramsay & Steel (1985) ▶; Samaritoni *et al.* (2007 ▶). For graph-set notation, see: Bernstein *et al.* (1995 ▶).
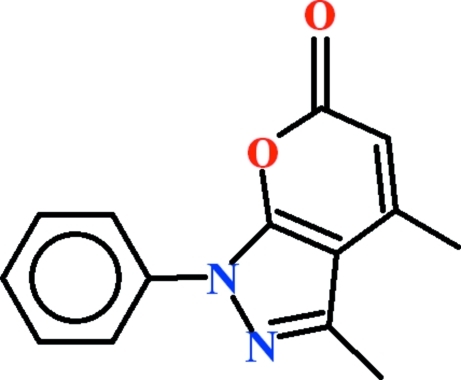


## Experimental

### 

#### Crystal data

C_14_H_12_N_2_O_2_
*M* *_r_* = 240.26
Monoclinic, 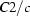

*a* = 15.1231 (9) Å
*b* = 13.3558 (8) Å
*c* = 13.8684 (8) Å
β = 120.965 (2)°
*V* = 2401.9 (3) Å^3^
*Z* = 8
Mo *K*α radiation
μ = 0.09 mm^−1^
*T* = 296 K
0.35 × 0.25 × 0.25 mm

#### Data collection

Bruker Kappa APEXII CCD diffractometer
Absorption correction: multi-scan (*SADABS*; Bruker, 2005 ▶) *T*_min_ = 0.975, *T*_max_ = 0.982
9214 measured reflections
2171 independent reflections
1382 reflections with *I* > 2σ(*I*)
*R*_int_ = 0.032

#### Refinement

*R*[*F* ^2^ > 2σ(*F* ^2^)] = 0.046
*wR*(*F* ^2^) = 0.140
*S* = 1.02
2171 reflections
165 parameters
H-atom parameters constrained
Δρ_max_ = 0.15 e Å^−3^
Δρ_min_ = −0.19 e Å^−3^

### 

Data collection: *APEX2* (Bruker, 2009 ▶); cell refinement: *SAINT* (Bruker, 2009 ▶); data reduction: *SAINT*; program(s) used to solve structure: *SHELXS97* (Sheldrick, 2008 ▶); program(s) used to refine structure: *SHELXL97* (Sheldrick, 2008 ▶); molecular graphics: *ORTEP-3 for Windows* (Farrugia, 1997 ▶) and *PLATON* (Spek, 2009 ▶); software used to prepare material for publication: *WinGX* (Farrugia, 1999 ▶) and *PLATON*.

## Supplementary Material

Crystal structure: contains datablocks global, I. DOI: 10.1107/S1600536811011317/hb5827sup1.cif

Structure factors: contains datablocks I. DOI: 10.1107/S1600536811011317/hb5827Isup2.hkl

Additional supplementary materials:  crystallographic information; 3D view; checkCIF report

## Figures and Tables

**Table 1:** Hydrogen-bond geometry (Å, °) *Cg*3 is the centroid of the C1–C6 phenyl ring.

*D*—H⋯*A*	*D*—H	H⋯*A*	*D*⋯*A*	*D*—H⋯*A*
C3—H3⋯O2^i^	0.93	2.51	3.407 (3)	163
C6—H6⋯O1	0.93	2.29	2.938 (3)	126
C14—H14*C*⋯*Cg*3^ii^	0.96	2.75	3.506 (2)	136

Symmetry codes: (i) 
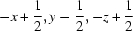
; (ii) 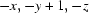.

**Fig. 1.**
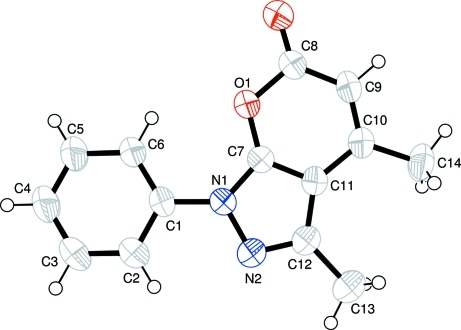
View of (I) with displacement ellipsoids drawn at the 50% probability level.

**Fig. 2.**
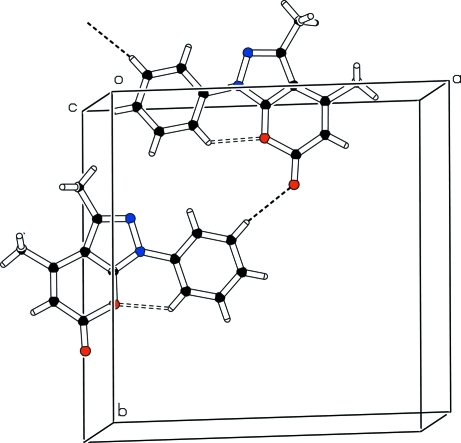
Packing diagram of the title compound showing that polymeric chains are formed.

## supplementary crystallographic information

### Comment

The parent ring system pyrano[2,3-*c*]pyrazol-6-one is an isostere of coumarin. A number of its derivatives have been prepared from corresponding hydrazines and beta ketoesters (Khan *et al.*, 1982, Samaritoni *et al.*, 2007). It has been shown that these contain analgesic and anti-inflammatory activities (Kuo *et al.*, 1984, Abdallah & Zaki, 1999) and while others are tested for their antiplatelet activity (Huang *et al.*, 1992). The title compound (I, Fig. 1) has been synthesized and its crystal structure is being reported here.

The crystal structure of (II) *i.e.*, 3,4-dimethyl-1-(2-pyridyl)pyrano(2,3 - c)pyrazol-6(1*H*)-one (Ramsay & Steel, 1983) has been published which differs from (I) due to pyridal attachment instead of phenyl and hence is closely related.

In (I) the phyenyl ring A (C1–C6) and 3,4-dimethylpyrano[2,3-*c*]pyrazol -6(1*H*)-one moiety are almost planar with r. m. s. deviations of 0.003 and 0.023 Å, respectively. The dihedral angle between A/B is 7.28 (6)°. The molecules are linked by C(8) polymeric chains (Bernstein *et al.*, 1995) due to H-boning of C—H···O type (Table 1, Fig. 2). An intramolecular H-bonding and a C—H···π interaction (Table 1) also play an important role in stabilizing the molecules. There also exist π···π interactions between the pyrazole rings at a distance of 3.6374 (12) Å.

### Experimental

A mixture of 3-methyl-1-phenylpyrazol-5-one (17.4 g, 0.1 mol) and ethyl acetoacetate (13 g, 0.1 mol) was heated at 413 K (oil bath) for an hour, cooled and triturated with 200 ml of pet. ether (bp. 313–333 K) and filtered to give the title compound.  Light brown rods of (I) were obtained on recrystallization from ethanol. Yield 10.8 g, 45%: m.p. 415 K

### Refinement

The H-atoms were positioned geometrically (C–H = 0.93–0.96 Å) and refined as riding with *U*_iso_(H) = x*U*_eq_(C), where x = 1.5 for methyl and x = 1.2 for aryl H-atoms.

### Crystal data

**Table:**

C_14_H_12_N_2_O_2_	*F*(000) = 1008
*M**_r_* = 240.26	*D*_x_ = 1.329 Mg m^−^^3^
Monoclinic, *C*2/*c*	Mo *K*α radiation, λ = 0.71073 Å
Hall symbol: -C 2yc	Cell parameters from 1382 reflections
*a* = 15.1231 (9) Å	θ = 2.2–25.3°
*b* = 13.3558 (8) Å	µ = 0.09 mm^−^^1^
*c* = 13.8684 (8) Å	*T* = 296 K
β = 120.965 (2)°	Rod, light brown
*V* = 2401.9 (3) Å^3^	0.35 × 0.25 × 0.25 mm
*Z* = 8	

### Data collection

**Table:**

Bruker Kappa APEXII CCD diffractometer	2171 independent reflections
Radiation source: fine-focus sealed tube	1382 reflections with *I* > 2σ(*I*)
graphite	*R*_int_ = 0.032
Detector resolution: 8.10 pixels mm^-1^	θ_max_ = 25.3°, θ_min_ = 2.2°
ω scans	*h* = −18→14
Absorption correction: multi-scan (*SADABS*; Bruker, 2005)	*k* = −15→16
*T*_min_ = 0.975, *T*_max_ = 0.982	*l* = −9→16
9214 measured reflections	

### Refinement

**Table:**

Refinement on *F*^2^	Primary atom site location: structure-invariant direct methods
Least-squares matrix: full	Secondary atom site location: difference Fourier map
*R*[*F*^2^ > 2σ(*F*^2^)] = 0.046	Hydrogen site location: inferred from neighbouring sites
*wR*(*F*^2^) = 0.140	H-atom parameters constrained
*S* = 1.02	*w* = 1/[σ^2^(*F*_o_^2^) + (0.0723*P*)^2^ + 0.5405*P*] where *P* = (*F*_o_^2^ + 2*F*_c_^2^)/3
2171 reflections	(Δ/σ)_max_ < 0.001
165 parameters	Δρ_max_ = 0.15 e Å^−^^3^
0 restraints	Δρ_min_ = −0.19 e Å^−^^3^

### Special details

**Table:**

Geometry. Bond distances, angles *etc*. have been calculated using the rounded fractional coordinates. All su's are estimated from the variances of the (full) variance-covariance matrix. The cell e.s.d.'s are taken into account in the estimation of distances, angles and torsion angles
Refinement. Refinement of *F*^2^ against ALL reflections. The weighted *R*-factor *wR* and goodness of fit *S* are based on *F*^2^, conventional *R*-factors *R* are based on *F*, with *F* set to zero for negative *F*^2^. The threshold expression of *F*^2^ > σ(*F*^2^) is used only for calculating *R*-factors(gt) *etc*. and is not relevant to the choice of reflections for refinement. *R*-factors based on *F*^2^ are statistically about twice as large as those based on *F*, and *R*- factors based on ALL data will be even larger.

### Fractional atomic coordinates and isotropic or equivalent isotropic displacement parameters (Å^2^)

**Table:**

	*x*	*y*	*z*	*U*_iso_*/*U*_eq_	
O1	0.01582 (9)	0.63464 (9)	0.13521 (10)	0.0584 (5)	
O2	−0.07464 (12)	0.77525 (12)	0.09062 (16)	0.0994 (7)	
N1	0.09619 (12)	0.47598 (11)	0.16813 (13)	0.0526 (6)	
N2	0.06513 (14)	0.37672 (12)	0.14972 (16)	0.0689 (7)	
C1	0.20381 (14)	0.49922 (15)	0.22105 (15)	0.0526 (7)	
C2	0.27219 (16)	0.42241 (17)	0.24254 (18)	0.0661 (8)	
C3	0.37688 (16)	0.44318 (19)	0.2968 (2)	0.0794 (9)	
C4	0.41168 (17)	0.5391 (2)	0.3279 (2)	0.0825 (9)	
C5	0.34341 (16)	0.61512 (19)	0.30539 (19)	0.0770 (9)	
C6	0.23861 (15)	0.59574 (16)	0.25142 (17)	0.0613 (8)	
C7	0.01255 (14)	0.53448 (14)	0.13093 (15)	0.0484 (7)	
C8	−0.08080 (15)	0.68528 (17)	0.08827 (18)	0.0659 (8)	
C9	−0.17132 (15)	0.62522 (17)	0.04570 (17)	0.0638 (8)	
C10	−0.17218 (14)	0.52456 (16)	0.04351 (15)	0.0537 (7)	
C11	−0.07438 (14)	0.47628 (14)	0.08801 (16)	0.0519 (7)	
C12	−0.03607 (17)	0.37748 (16)	0.10228 (19)	0.0657 (8)	
C13	−0.0944 (2)	0.28099 (17)	0.0706 (3)	0.1025 (13)	
C14	−0.27056 (15)	0.46714 (18)	−0.00325 (19)	0.0709 (8)	
H2	0.24847	0.35727	0.22090	0.0793*	
H3	0.42364	0.39162	0.31212	0.0953*	
H4	0.48198	0.55248	0.36445	0.0990*	
H5	0.36739	0.68028	0.32639	0.0924*	
H6	0.19215	0.64766	0.23586	0.0736*	
H9	−0.23416	0.65797	0.01749	0.0766*	
H13A	−0.04834	0.22680	0.08183	0.1536*	
H13B	−0.14832	0.28336	−0.00699	0.1536*	
H13C	−0.12397	0.27081	0.11674	0.1536*	
H14A	−0.32644	0.51265	−0.02276	0.1064*	
H14B	−0.26542	0.42052	0.05210	0.1064*	
H14C	−0.28305	0.43135	−0.06914	0.1064*	

### Atomic displacement parameters (Å^2^)

**Table:**

	*U*^11^	*U*^22^	*U*^33^	*U*^12^	*U*^13^	*U*^23^
O1	0.0380 (8)	0.0474 (9)	0.0769 (9)	0.0008 (6)	0.0203 (7)	−0.0016 (6)
O2	0.0515 (10)	0.0514 (11)	0.1565 (16)	0.0033 (8)	0.0259 (10)	−0.0053 (10)
N1	0.0409 (9)	0.0431 (10)	0.0687 (10)	0.0025 (8)	0.0245 (8)	0.0023 (7)
N2	0.0509 (12)	0.0454 (11)	0.1012 (13)	−0.0024 (8)	0.0325 (10)	0.0020 (9)
C1	0.0375 (11)	0.0555 (13)	0.0584 (11)	0.0036 (9)	0.0201 (9)	0.0015 (9)
C2	0.0503 (13)	0.0581 (14)	0.0807 (14)	0.0098 (11)	0.0272 (11)	0.0040 (10)
C3	0.0478 (14)	0.0753 (17)	0.1009 (17)	0.0200 (13)	0.0282 (13)	0.0069 (13)
C4	0.0374 (12)	0.0844 (18)	0.1021 (18)	0.0021 (13)	0.0191 (12)	−0.0092 (14)
C5	0.0428 (13)	0.0728 (16)	0.0992 (17)	−0.0039 (12)	0.0249 (12)	−0.0147 (13)
C6	0.0401 (12)	0.0550 (14)	0.0797 (13)	0.0014 (10)	0.0243 (10)	−0.0064 (10)
C7	0.0422 (11)	0.0434 (12)	0.0572 (11)	0.0013 (9)	0.0238 (9)	0.0009 (9)
C8	0.0431 (12)	0.0519 (14)	0.0856 (14)	0.0050 (11)	0.0208 (11)	−0.0010 (11)
C9	0.0357 (11)	0.0631 (15)	0.0793 (14)	0.0022 (10)	0.0201 (10)	−0.0039 (11)
C10	0.0411 (11)	0.0572 (13)	0.0587 (11)	−0.0032 (9)	0.0228 (9)	−0.0021 (9)
C11	0.0410 (11)	0.0496 (12)	0.0620 (12)	−0.0029 (9)	0.0244 (9)	−0.0002 (9)
C12	0.0525 (13)	0.0489 (14)	0.0905 (15)	−0.0057 (10)	0.0331 (12)	0.0013 (10)
C13	0.0688 (17)	0.0521 (16)	0.166 (3)	−0.0132 (13)	0.0457 (17)	−0.0009 (15)
C14	0.0444 (12)	0.0789 (16)	0.0838 (14)	−0.0161 (11)	0.0289 (11)	−0.0101 (12)

### Geometric parameters (Å, °)

**Table:**

O1—C7	1.339 (2)	C10—C11	1.430 (3)
O1—C8	1.427 (3)	C10—C14	1.493 (3)
O2—C8	1.204 (3)	C11—C12	1.414 (3)
N1—N2	1.386 (2)	C12—C13	1.494 (4)
N1—C1	1.433 (3)	C2—H2	0.9300
N1—C7	1.344 (3)	C3—H3	0.9300
N2—C12	1.319 (4)	C4—H4	0.9300
C1—C2	1.376 (3)	C5—H5	0.9300
C1—C6	1.374 (3)	C6—H6	0.9300
C2—C3	1.387 (4)	C9—H9	0.9300
C3—C4	1.368 (4)	C13—H13A	0.9600
C4—C5	1.365 (4)	C13—H13B	0.9600
C5—C6	1.385 (4)	C13—H13C	0.9600
C7—C11	1.372 (3)	C14—H14A	0.9600
C8—C9	1.426 (3)	C14—H14B	0.9600
C9—C10	1.345 (3)	C14—H14C	0.9600
			
C7—O1—C8	116.58 (17)	N2—C12—C13	119.9 (2)
N2—N1—C1	119.31 (17)	C11—C12—C13	128.8 (3)
N2—N1—C7	108.83 (18)	C1—C2—H2	120.00
C1—N1—C7	131.86 (16)	C3—C2—H2	120.00
N1—N2—C12	106.32 (18)	C2—C3—H3	120.00
N1—C1—C2	118.57 (18)	C4—C3—H3	120.00
N1—C1—C6	121.0 (2)	C3—C4—H4	120.00
C2—C1—C6	120.4 (2)	C5—C4—H4	120.00
C1—C2—C3	119.3 (2)	C4—C5—H5	120.00
C2—C3—C4	120.3 (2)	C6—C5—H5	120.00
C3—C4—C5	120.1 (3)	C1—C6—H6	120.00
C4—C5—C6	120.4 (2)	C5—C6—H6	120.00
C1—C6—C5	119.5 (2)	C8—C9—H9	118.00
O1—C7—N1	123.92 (19)	C10—C9—H9	118.00
O1—C7—C11	126.2 (2)	C12—C13—H13A	109.00
N1—C7—C11	109.87 (17)	C12—C13—H13B	109.00
O1—C8—O2	114.4 (2)	C12—C13—H13C	109.00
O1—C8—C9	117.49 (19)	H13A—C13—H13B	109.00
O2—C8—C9	128.1 (2)	H13A—C13—H13C	109.00
C8—C9—C10	124.6 (2)	H13B—C13—H13C	109.00
C9—C10—C11	116.4 (2)	C10—C14—H14A	109.00
C9—C10—C14	121.3 (2)	C10—C14—H14B	109.00
C11—C10—C14	122.28 (19)	C10—C14—H14C	109.00
C7—C11—C10	118.64 (18)	H14A—C14—H14B	109.00
C7—C11—C12	103.7 (2)	H14A—C14—H14C	109.00
C10—C11—C12	137.7 (2)	H14B—C14—H14C	109.00
N2—C12—C11	111.3 (2)		
			
C8—O1—C7—N1	177.83 (18)	C1—C2—C3—C4	−0.5 (4)
C8—O1—C7—C11	−1.5 (3)	C2—C3—C4—C5	−0.2 (4)
C7—O1—C8—O2	−177.76 (19)	C3—C4—C5—C6	0.3 (4)
C7—O1—C8—C9	2.7 (3)	C4—C5—C6—C1	0.3 (3)
C1—N1—N2—C12	179.03 (18)	O1—C7—C11—C10	−0.4 (3)
C7—N1—N2—C12	−0.1 (2)	O1—C7—C11—C12	179.28 (19)
N2—N1—C1—C2	5.9 (3)	N1—C7—C11—C10	−179.82 (17)
N2—N1—C1—C6	−173.10 (19)	N1—C7—C11—C12	−0.1 (2)
C7—N1—C1—C2	−175.1 (2)	O1—C8—C9—C10	−2.2 (3)
C7—N1—C1—C6	5.8 (3)	O2—C8—C9—C10	178.3 (2)
N2—N1—C7—O1	−179.24 (17)	C8—C9—C10—C11	0.3 (3)
N2—N1—C7—C11	0.2 (2)	C8—C9—C10—C14	−179.6 (2)
C1—N1—C7—O1	1.7 (3)	C9—C10—C11—C7	1.0 (3)
C1—N1—C7—C11	−178.87 (19)	C9—C10—C11—C12	−178.6 (2)
N1—N2—C12—C11	0.1 (2)	C14—C10—C11—C7	−179.04 (19)
N1—N2—C12—C13	179.6 (2)	C14—C10—C11—C12	1.4 (4)
N1—C1—C2—C3	−178.0 (2)	C7—C11—C12—N2	0.0 (3)
C6—C1—C2—C3	1.0 (3)	C7—C11—C12—C13	−179.5 (3)
N1—C1—C6—C5	178.11 (19)	C10—C11—C12—N2	179.6 (2)
C2—C1—C6—C5	−0.9 (3)	C10—C11—C12—C13	0.1 (5)

### Hydrogen-bond geometry (Å, °)

**Table:**

Cg3 is the centroid of the C1–C6 phenyl ring.

**Table:**

*D*—H···*A*	*D*—H	H···*A*	*D*···*A*	*D*—H···*A*
C3—H3···O2^i^	0.93	2.51	3.407 (3)	163
C6—H6···O1	0.93	2.29	2.938 (3)	126
C14—H14C···Cg3^ii^	0.96	2.75	3.506 (2)	136

Symmetry codes: (i) −*x*+1/2, *y*−1/2, −*z*+1/2; (ii) −*x*, −*y*+1, −*z*.
